# Renal replacement therapy practices for patients with acute kidney injury in China

**DOI:** 10.1371/journal.pone.0178509

**Published:** 2017-07-10

**Authors:** William R. Clark, Xiaoqiang Ding, Haibo Qiu, Zhaohui Ni, Ping Chang, Ping Fu, Jiarui Xu, MinMin Wang, Li Yang, Jing Wang, Claudio Ronco

**Affiliations:** 1 Davidson School of Chemical Engineering, Purdue University, West Lafayette (IN), United States of America; 2 Department of Nephrology, Zhongshan Hospital, Fudan University, Shanghai, China; 3 Shanghai Institute of Kidney Disease and Dialysis, Shanghai Quality Control Center for Dialysis, Shanghai, China; 4 Department of Critical Care Medicine, Nanjing Zhongda Hospital, Southeast University, Nanjing, China; 5 Department of Nephrology, Renji Hospital, Shanghai Jiao Tong University School of Medicine, Shanghai, China; 6 Department of ICU, Zhujiang Hospital, Southern Medical University, Guangzhou, China; 7 Division of Nephrology, West China Hospital of Sichuan University, Chengdu, China; 8 Baxter Healthcare, Shanghai, China; 9 Guanlan Networks CO., LTD., Hangzhou, China; 10 International Renal Research Institute of Vicenza (IRRIV), San Bortolo Hospital, Vicenza, Italy; 11 Department of Nephrology, San Bortolo Hospital, Vicenza, Italy; Bambino Gesù Children's Hospital, ITALY

## Abstract

Recent data indicate AKI is very common among hospitalized Chinese patients and continuous renal replacement therapy (CRRT) is increasingly offered for treatment. However, only anecdotal information regarding CRRT’s use in relation to other modalities and the specific manner in which it is prescribed exists currently. This report summarizes the results of a comprehensive physician survey designed to characterize contemporary dialytic management of AKI patients in China, especially with respect to the utilization of CRRT. The survey queried both nephrologists and critical care physicians across a wide spectrum of hospitals about factors influencing initial RRT modality selection, especially patient clinical characteristics and willingness to receive RRT, treatment location, and institutional capabilities. For patients initially treated with CRRT, data related to indication, timing of treatment initiation, dose, anticoagulation technique, and duration of therapy were also collected. Among AKI patients considered RRT candidates, the survey indicated 15.1% (95% CI, 12.3%-17.9%) did not actually receive dialysis at Chinese hospitals. The finding was largely attributed to prohibitively high therapy costs in the view of patients or their families. The survey confirmed the dichotomy in RRT delivery in China, occurring both in the nephrology department (with nephrologists responsible) and the intensive care unit (with critical care physicians responsible). For all patients who were offered and received RRT, the survey participants reported 63.9% (56.4%-71.3%) were treated initially with CRRT and 24.8% (19.2%-30.3%) with intermittent hemodialysis (HD) (P<0.001). The mean percentage of patients considered hemodynamically unstable at RRT initiation was 36.2% (31.3%-41.1%), although this figure was two-fold higher in patients treated initially with CRRT (43.1%; 35.8%-50.4%) in comparison to those initially treated with HD (22.4%; 16.4%-28.4%)(P<0.001). An overwhelming majority of intensive care patients were treated initially with CRRT (86.6%; 79.8–93.4%) while it was the initial modality in only 44.6% (33.5–55.7%) of patients treated in a nephrology department (P<0.001). Approximately 70% of respondents overall reported prescribing a CRRT dose in the range of 20–30 mL/kg/hr while approximately 20% of prescriptions fell above this range. Daily prescribed therapy duration demonstrated a marked divergence from values reported in the literature and standard clinical practice. Overall, the most common average prescribed value (50% of respondents) fell in the 10–20 hr range, with only 18% in the 20–24 hr range. Moreover, 32% of respondents reported an average prescribed value of less than 10 hrs per day. While the percentages for the 10–20 hrs range were essentially the same for nephrology and ICU programs, a daily duration of less than 10 hrs was much more common in nephrology programs (48.0%; 38.3%-57.9%) versus ICU programs (16%; 10.0%-24.6%)(P<0.001). Our analysis demonstrates both similarities and differences between RRT practices for AKI in China and those in the developed world. While some differences are driven by non-medical factors, future studies should explore these issues further as Chinese RRT practices are harmonized with those in the rest of the world.

## Introduction

In the developed world, clinicians offer renal replacement therapy to the vast majority of acute kidney injury (AKI) patients having a valid indication, even as the incidence of this disorder continues to grow [**1**]. However, the specific choice of renal replacement modality for critically ill patients with AKI remains controversial [**2**], being influenced by not only a patient's clinical condition but also the knowledge and experience of both the prescribing clinician and institution [**3**]. Although continuous renal replacement therapy (CRRT) is now a mainstay therapy in the intensive care unit (ICU) and its utilization continues to increase on a global basis [**4**], its application in clinical practice is quite variable, even in geographic regions in which widespread acceptance of the therapy already exists [**5**]. This clinical practice variation is even more pronounced in the developing world, in which resource constraints, RRT costs, and physician acceptance are important considerations [**6**].

As the world’s most populous country, China is experiencing rapid growth in the utilization of medical technology [**7**]. Recent data indicate AKI is very common among hospitalized Chinese patients [**8–11**] and CRRT is one technology that is increasingly being offered [**12**]. However, only anecdotal information regarding CRRT’s use in relation to other modalities and the specific manner in which it is prescribed exists currently [**13**].

This report summarizes the results of a comprehensive physician survey designed to characterize contemporary dialytic management of AKI patients in China, especially with respect to the utilization of CRRT. For the survey, 100 nephrologists and 100 critical care physicians across the spectrum of locale, hospital type, and clinical experience were queried. The survey focused on the factors influencing initial RRT modality selection, especially patient clinical characteristics and willingness to receive RRT, treatment location, and institutional capabilities. For patients initially treated with CRRT, data related to indication, timing of treatment initiation, dose, anticoagulation technique, and duration of therapy were also collected.

## Methods

### Description of survey and participants

The survey was conducted on the platform of DXY.cn, the largest online community for physicians and health care professionals in China with more than 3.2 million members [**14**]. We excluded doctors from departments other than nephrology or critical care, along with those from smaller hospitals without CRRT equipment and those without CRRT experience. The online digital questionnaire was distributed to 200 physicians between March 16, 2016 and June 12, 2016 and focused on the initial RRT modality prescribed for AKI patients.

One of the strict requirements of the survey was the inclusion of only one physician from the same department of a particular hospital. The survey data were analyzed based on the assumption that responses from a physician practicing in a specific department of a given hospital were representative of all the physicians in that department with regard to renal replacement therapy. In turn, this assumption was predicated upon the observation that most institutions employ RRT protocols that largely standardize local clinical practice.

The Chinese physicians participating in the survey were chosen with the goal of achieving equal distribution with respect to sub-specialty (nephrology and critical care), hospital type (Class 2 and 3) [**15**], and city size (Tier 1, Tier 2, and Tier 3)[**16**]. *Class 2* hospitals are typically found in cities of medium size and have between 100 and 500 beds—in addition to providing healthcare, they are usually also engaged in medical education and research on a regional basis. *Class 3* hospitals are large institutions (more than 500 beds) that provide both general and specialist care from a large referral base while also actively supporting medical education and research. Although strict definitions do not exist, the following classification of cities is generally accepted: 1) *Tier 1*: four largest cities in China (Beijing, Shanghai, Guangzhou, and Shenzhen); 2) *Tier 2*: large provincial capitals and coastal cities, such as Xiamen, Chongqing, Chengdu, and Wuhan; and 3) *Tier 3*: provincial cities of medium size.

In Chinese clinical practice, the location of RRT delivery has been dependent historically on the responsible physician. Critical care physicians provide RRT (predominantly CRRT) in a typical medical or surgical ICU. On the other hand, both CRRT and intermittent modalities are managed by nephrologists outside an ICU environment in a hospital ward having an embedded dialysis unit. Understanding the manner in which the location of care influences RRT prescription and delivery was a major objective of this study.

The study was conducted using an online questionnaire. Invitations to participate in the study were sent randomly to 1,200 physician members of DXY.cn by email, which described the purpose of the study. Members who were interested in participating were apprised further of the study through an email link. The link provided the interested members the opportunity to confirm their agreement to continue and complete the questionnaire. The participants were enrolled sequentially in the survey on a “first-come, first-serve” basis, with the primary criteria being equal numbers of nephrologists and critical care physicians and equal distribution of participants from Tier 1, 2, and 3 cities.

Upon agreeing to participate, the DXY member was provided a unique study identification number. This unique number was linked anonymously to a respondent’s membership information in the DXY main database, to which only an authorized DXY team member had access. DXY policy strictly forbids sharing member data with a third-party and the study sponsor had no access to this information. Moreover, the questionnaire itself contained no questions regarding respondents' private information, such as name, email address, phone number, or institution.

The respondents in the study agreed to participate with knowledge that the results of the survey would be analyzed by the sponsor and published. The input provided by the participants reflected their general experience in the clinical management of AKI patients and no patient specific-data were provided at any point during the study. Based on this operational structure, a decision was made by the sponsor and DXY to waive the requirement for ethical committee/institutional review board approval at the regional or institutional level.

### General assessment of RRT usage

Participants were queried regarding the following: 1) number of beds and CRRT machines in their department; 2) number of incident AKI patients having an indication for RRT per month in their department and the percentage of such patients who do not receive RRT; 3) percentage of hemodynamically unstable patients of those who receive RRT; and 4) percentage of AKI patients treated initially with CRRT, intermittent hemodialysis (HD), sustained low-efficiency dialysis (SLED), and peritoneal dialysis (PD).

The classification of a particular prescription was based on the type of machine used. A treatment was designated as “CRRT” if a dedicated device for this modality was used, irrespective of the prescribed time. On the other hand, all other extracorporeal treatments were delivered with a standard hemodialysis machine. Information about estimated percent delivery of the prescribed dose was not obtained in the survey.

### Assessment of RRT management

Questions regarding RRT management preferences included indications for RRT initiation, timing of RRT initiation, and reasons for choosing a specific RRT modality. Timing of RRT initiation was further divided into the specific criteria and their thresholds, including serum creatinine, blood urea nitrogen, blood potassium, oliguria, and fluid overload. Other questions posed to the survey participants related to RRT complications, key factors in AKI survival and kidney recovery, and patient follow-up strategies.

Questions specific to CRRT prescription preferences focused on modality, dose, anticoagulant, and both overall and daily treatment duration. Finally, the survey participants were asked to provide the main challenges faced in CRRT implementation. The questionnaire in its entirety appears in **S1 Table**.

### Statistical analysis

Results are expressed as mean±SD for continuous variables, and frequency (percentage) for categorical variables. Differences between nephrology and critical care responses were assessed using t-tests for continuous variables and by χ^2^ tests for categorical variables. The RRT modality distributions (CRRT, HD, SLED, and PD) are expressed as percentages (95% confidence interval). All statistical analyses were performed using STATA statistical software (version 14.0; Statacorp, College Station, TX).

## Results

### General findings

#### Characteristics of survey participants

From a seniority perspective, the majority of participants (50%) fell in the “attending” category and approximately two-thirds of physicians in both the nephrology and critical care groups had at least three years of experience in managing CRRT patients. Not surprisingly, CRRT experience also was associated with seniority as nearly 90% of physicians at the vice chief/chief level but only 21% of residents reported three years or more of CRRT experience. Reflecting a broad spectrum of viewpoints, physicians practicing at 189 different hospitals across 81 cities and 26 provinces in China participated in the survey.

The goals of enrolling equal numbers of nephrologists and critical care physicians with an equal distribution of participants from Tier 1, 2, and 3 cities were achieved. Although an additional goal was an equal distribution of participants from Class 2 and 3 hospitals, this was not achieved. Nevertheless, the distribution actually achieved in the survey (30% and 70% of respondents from Class 2 and 3 hospitals, respectively) generally reflects AKI clinical practice in China.

The characteristics of the survey participants are summarized in **Table 1.**

**Table 1:** Characteristics of survey participants.

	Tier 1	Tier 2	Tier 3
	34%	34%	33%
**Hospital Class**	**Class 3**	**Class 2**	
	70%	30%	
**Department**	**ICU**	**Nephrology**	
	50%	50%	
**Job title**	**Vice/Chief**	**Attending**	**Resident**
	30%	50%	20%
**CRRT experience**	**<1 year**	**1–3 years**	**>3 years**
**ICU**	13%	19%	68%
**Nephrology**	9%	30%	61%
**Class 3**	9%	24%	67%
**Class 2**	15%	27%	58%
**Vice/Chief**	2%	10%	88%
**Attending**	13%	21%	66%
**Resident**	21%	56%	23%

#### Factors influencing provision of renal replacement therapy

For the departments at which the survey participants practiced, the mean monthly number of AKI patients having an indication for RRT was 7.7±8.1 while the mean number of hospital beds available for RRT was 35.8±32.4. Both of these parameters were highest among hospitals in Tier 2 cities. With respect to hospital type, the number of hospital beds and AKI patients was approximately 60% and 80% greater, respectively, in Class 3 vs Class 2 hospitals. Thus, the most common clinical scenario for RRT provision was a Class 3 hospital in a Tier 2 city.

For AKI patients considered RRT candidates, the survey participants estimated that 15.1% (95% CI, 12.3%-17.9%) ultimately did not receive dialysis. The finding was attributed largely to prohibitively high therapy costs in the view of patients or their families. Other explanations reported for this finding include advanced age, futility, and inadequate RRT training of the clinical care team.

#### Modalities and location of renal replacement therapy

**Fig 1:**
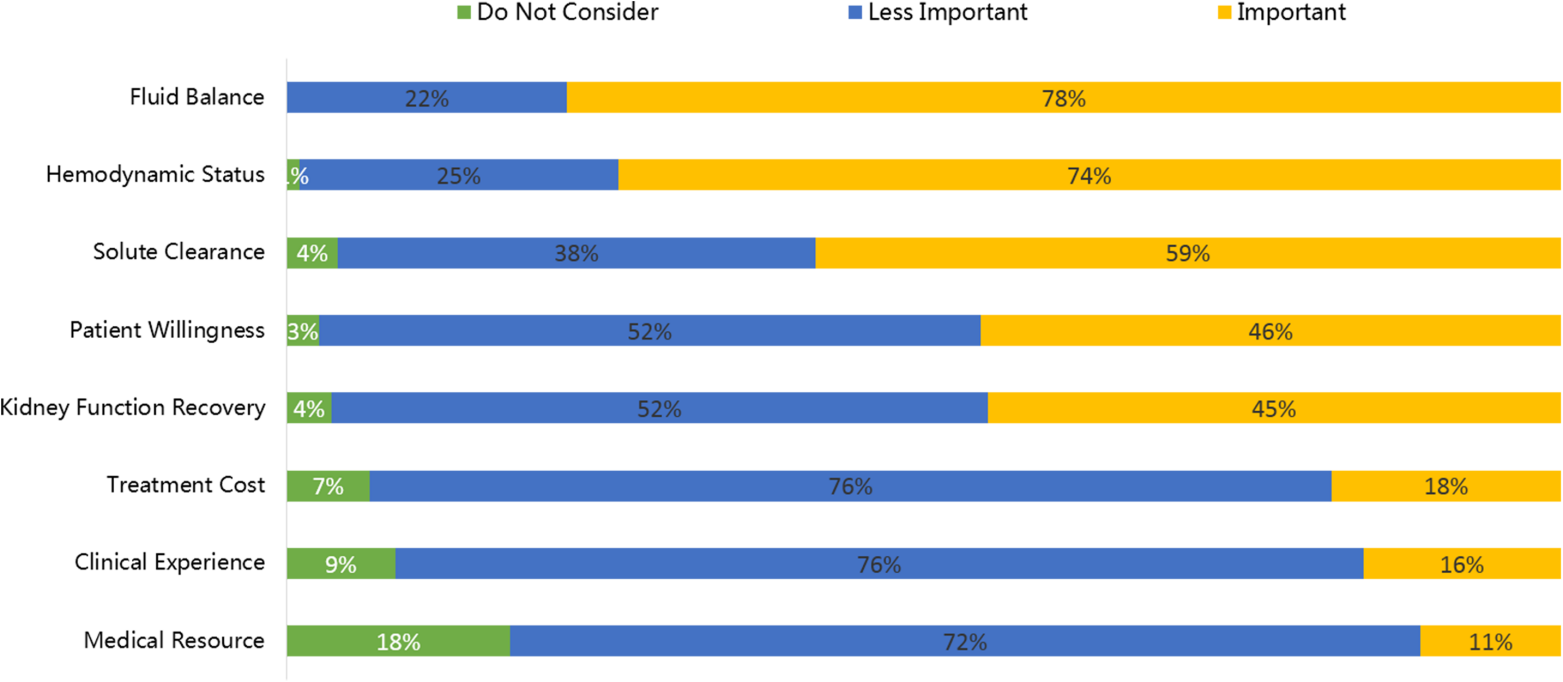
Most important factors influencing initial RRT modality choice for Chinese physicians.

The survey participants were asked to rank the most important factors influencing their decisions about RRT modality. As shown in **Fig 1**, fluid balance and hemodynamic stability were the most commonly listed criteria while solute clearance and renal recovery also received relatively high rankings. On the other hand, numerous non-medical factors were also considered important, including patient willingness and treatment cost.

**Fig 2:**
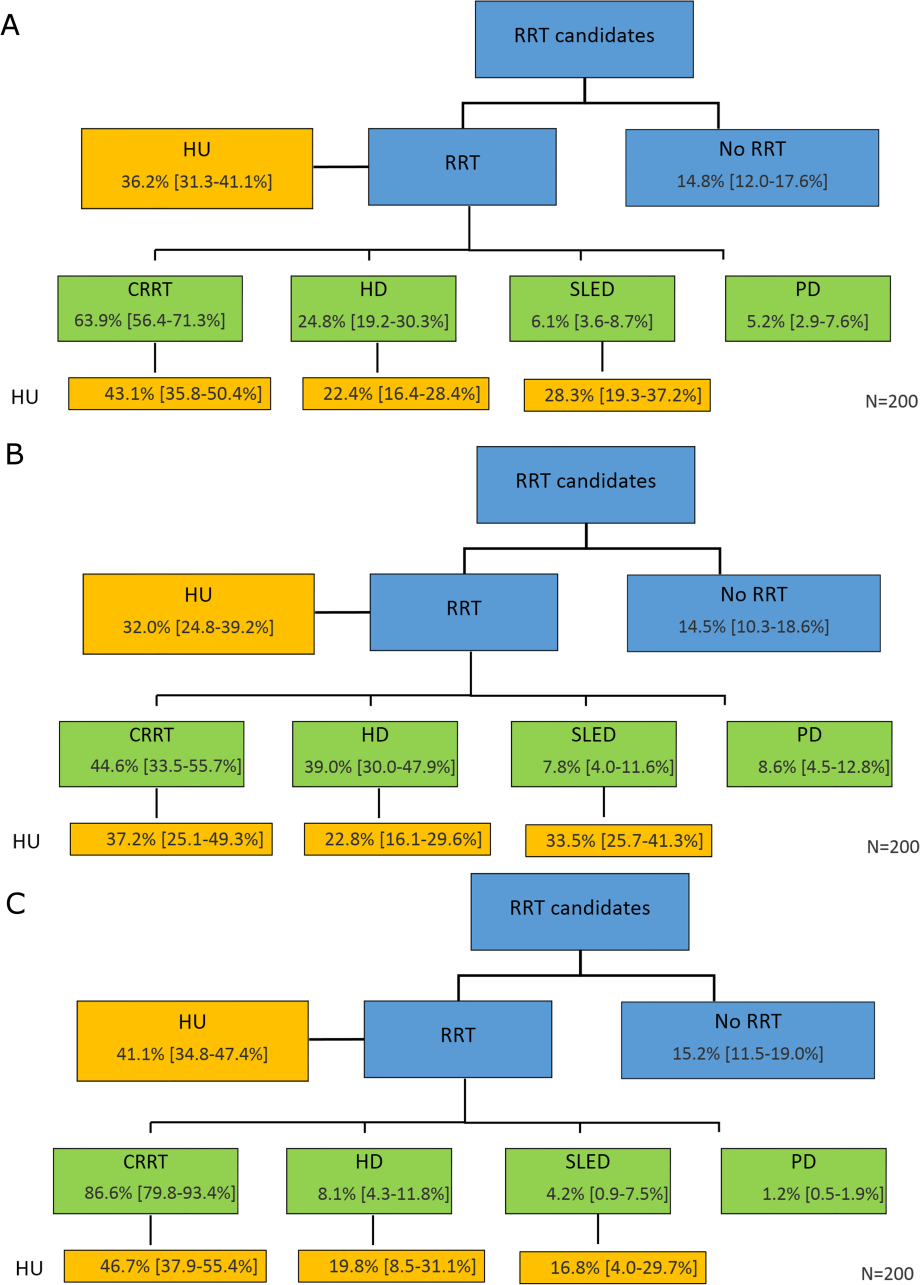
Initial RRT modality for Chinese AKI patients. A) All patients; B) Patients managed in the ICU; and C) Patients managed in the nephrology department. (HU = hemodynamically unstable). Results are expressed as mean [95% confidence interval].

For all patients who received RRT, the survey participants reported 63.9% (56.4%-71.3%) were treated initially with CRRT and 24.8% (19.2%-30.3%) with HD (P<0.001)–SLED and PD were the initial treatment for the remaining small percentage of patients (**Fig 2A**). The mean percentage of patients considered hemodynamically unstable at RRT initiation was 36.2% (31.3%-41.1%), although this figure was two-fold higher in patients treated initially with CRRT (43.1%; 35.8%-50.4%) versus HD (22.4%; 16.4%-28.4%)(P<0.001).

Differences emerge when the location of care (nephrology ward versus ICU) is considered. An overwhelming majority of ICU patients were treated initially with CRRT (86.6%; 79.8%-93.4%)(**Fig 2B)** while it was the initial modality in 44.6% (33.5%-55.7%) of patients treated in a nephrology department (P<0.001) (**Fig 2C)**. While this finding can be partly explained by the generally greater degree of hemodynamic instability among the ICU patients, a significant percentage of ICU patients treated with CRRT were not categorized as hemodynamically unstable, as mentioned above.

On the other hand, initial RRT modality application was more evenly distributed when treatment was provided in the nephrology department (**Fig 2C**). In this care environment, a relatively small percentage of patients initially treated with both CRRT (37.2%; 25.1%-49.3%) and HD (22.8%; 16.1%-29.6%)(P<0.01) were considered hemodynamically *unstable*.

#### Initiation of renal replacement therapy

The generic term “acute kidney injury” was reported by 71% of survey participants as an important factor for RRT initiation while electrolyte/acid-base disturbances, multi-organ failure, and fluid overload were also mentioned prominently. However, the specific importance of azotemia as a RRT trigger was uncertain, as 30–50% of the respondents did not routinely incorporate serum creatinine or urea in their analysis.

Nearly two-thirds of the respondents considered oliguria (criterion: 0.5 mL/kg/hr) for a period of at least 12 hrs to be an important RRT initiation criterion. Critical care physicians in general reported a lower serum creatinine and oliguria threshold than did nephrologists for RRT initiation. Specifically, 37% (28.0%-47.0%) of critical care physicians considered 6 hrs of oliguria to be a reasonable indication for RRT initiation versus 18% (11.6%-29.6%) of nephrologists (P<0.01).

On the other hand, the survey responses regarding fluid overload as a potential RRT initiation criterion presented a mixed picture, as nearly 20% of physicians did not believe percent fluid overload (%FO) is a relevant consideration at all. Moreover, only 5% of the respondents considered a threshold %FO value of 10% to be a valid initiation criterion while 35% and 42% reported using threshold %FO values of 15% and 20%, respectively.

#### Survival and renal recovery

**Fig 3:**
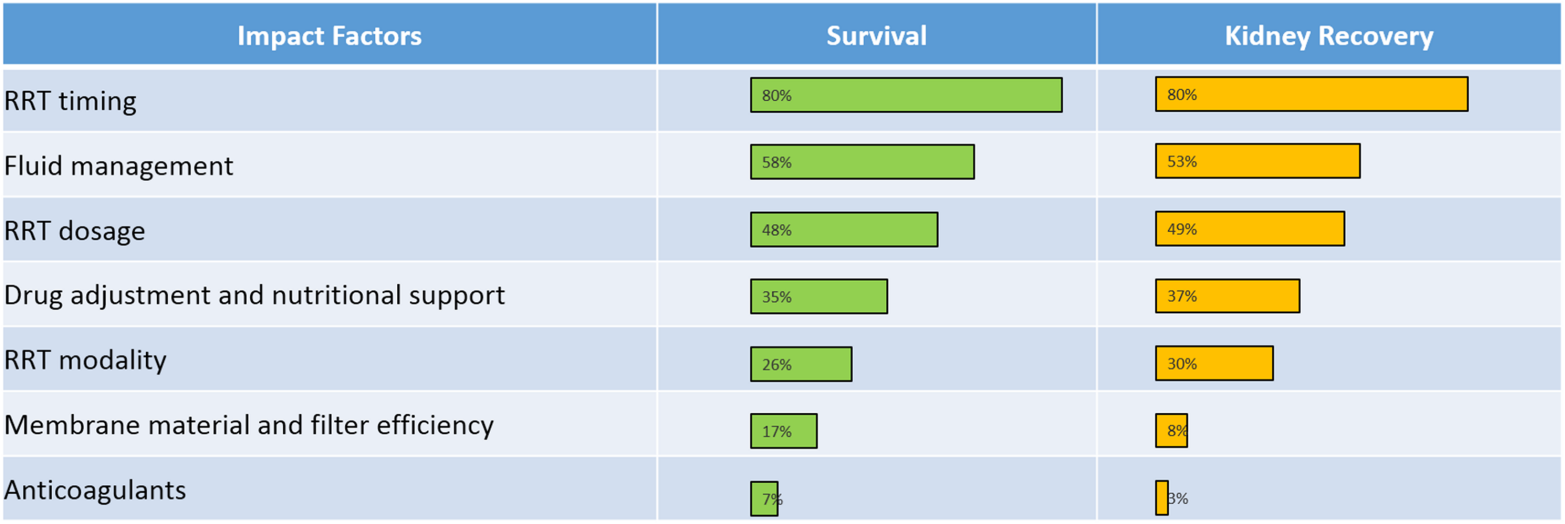
Factors listed by Chinese physicians as important in influencing survival and renal recovery.

In general, the factors listed by the survey participants as important in influencing survival and renal recovery tended to correlate (**Fig 3**). Timing of RRT initiation was the most important factor felt to influence both of these outcomes, being listed by 80% of participants in each case. Likewise, fluid management was considered important for both survival (58%) and renal recovery (53%), as was RRT dose. Less commonly reported factors, all listed by less than 50% of the participants, included drug dosing, nutritional support, RRT modality, filter/membrane characteristics, and anticoagulation.

Overall, patient disposition after discharge was approximately equally distributed according to: 1) follow-up specifically for the AKI episode; 2) follow-up for the primary disease; and 3) no follow-up. However, nearly half of the nephrology respondents reported regular follow-up specifically for the AKI episode while only a quarter of ICU respondents did so. Nearly half of the ICU respondents reported no regular follow-up at all after severe AKI.

### Findings specific to CRRT

#### CRRT utilization

For the institutions involved in the survey, the mean number of CRRT machines per hospital was 2.9±4.1. However, CRRT capacity varied considerably according to city tier and hospital class. For Tier 1, 2, and 3 cities, these median values were 2.8±2.5, 4.1±6.5, and 1.9±1.1 (P<0.001), respectively, while they were 1.6±1.1 and 3.5±4.8 in Class 2 and 3 hospitals (P<0.005), respectively. These data for CRRT reflect the overall RRT utilization data mentioned above. However, in all combinations of city size/hospital class, more CRRT machines were found in nephrology departments than ICUs. These differences were particularly pronounced in Tier 2 cities (median, 5.6±8.5 vs 2.4±1.8 CRRT machines per hospital; nephrology vs ICU; P<0.05) and Class 3 hospitals (median, 4.3±6.2 vs 2.6±2.2 CRRT machines per hospital; nephrology vs ICU; P<0.05).

#### CRRT treatment characteristics and complications

Overall CVVH was the most commonly prescribed modality (42%), followed by CVVHDF (35%) and CVVHD (23%). However, these preferences again segregated according to sub-specialty. For CRRT programs managed by ICUs, convective modalities (CVVH and CVVHDF) accounted for more than 80% of the responses. On the other hand, modality was essentially evenly distributed among nephrology-led programs. Approximately 70% of respondents overall reported prescribing a CRRT dose in the range of 20–30 mL/kg/hr while approximately 20% of prescriptions fell above this range.

Heparin-based anticoagulation for CRRT was prescribed by two-thirds of the respondents while one quarter prescribed regional citrate anticoagulation (RCA) and a distinct minority (10%) no anticoagulation. However, differences between nephrology and critical care practices were evident. Among critical care respondents, 32.0% (16.6%-47.4%) prescribed RCA while only 18.6% (11.0%-26.1%) prescribed low-molecular weight heparin (LMWH) (P = NS). On the other hand, 52.0% (39.1%-65.0%) of nephrologists prescribed LMWH while only 13.7% (3.0%-24.3%) prescribed RCA (P<0.001).

**Fig 4:**
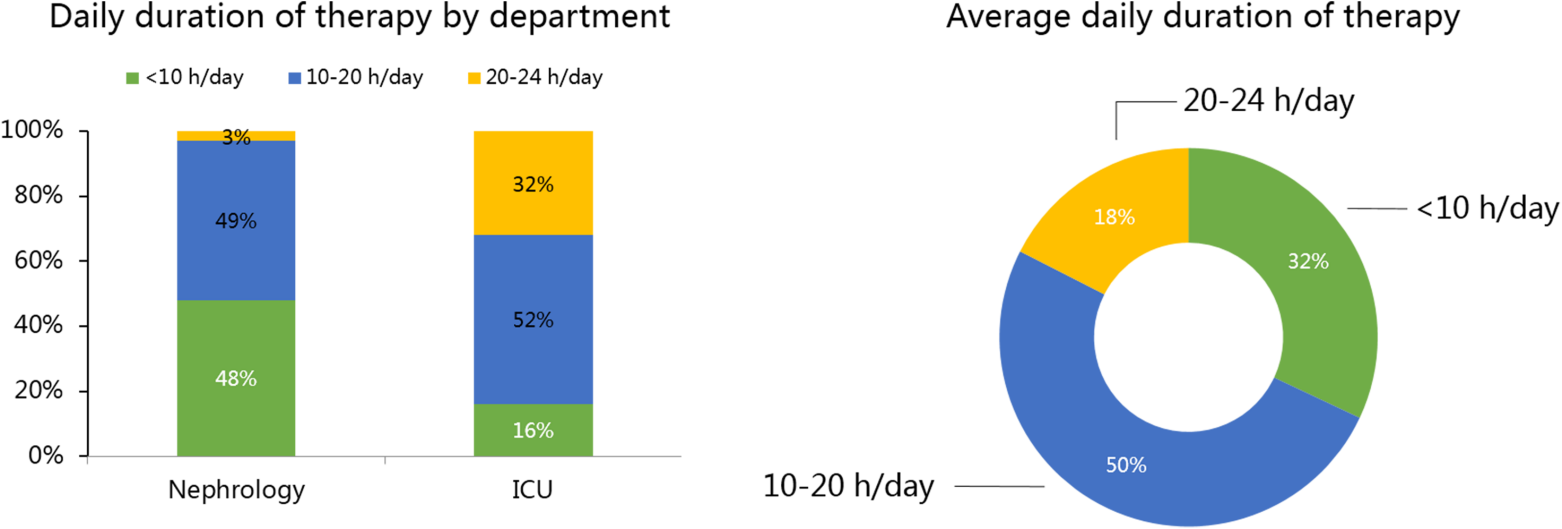
Prescribed daily duration of CRRT in China. A) According to department; B) All physicians.

A clear majority of treatments (71%) were reported typically to have an *overall* duration of 3–7 days. With respect to *daily* prescribed therapy duration, the most common average prescribed value (50% of all respondents) fell in the 10–20 hr range, with only 18% in the 20–24 hr range. Moreover, 32% of respondents reported an average prescribed value of less than 10 hrs per day. While the percentages for the 10–20 hrs range were essentially the same for nephrology and ICU programs, a daily duration of less than 10 hrs was much more common in nephrology programs (48.0%; 38.3%-57.9%) versus ICU programs (16%; 10.0%-24.6%)(P<0.001). (**Fig 4**).

With respect to complications, the survey participants were asked to rate as “important”, “less important”, or “not important” the following six complications: 1) hemorrhage and thrombosis; 2) filter clotting; 3) hypotension; 4) catheter complications; 5) drug clearance; and 6) membrane allergy. Hemorrhage/thrombosis, filter clotting, and hypotension were ranked by approximately half of the respondents as “important” while catheter complications and drug clearance received the same designation by approximately 25%. On the other hand, only 7% felt membrane allergy to be “important” and 50% considered it “not important”.

#### Potential barriers to CRRT utilization

The cost of therapy from the patient’s perspective was judged to be the most serious challenge facing CRRT use in China, being listed by 61% of respondents. The lack of clear markers for both treatment efficiency and therapy initiation was also cited by more than half the participants. Less commonly reported barriers were the lack of specific treatment guidelines, inadequate CRRT training for healthcare professionals, and insufficient institutional resources to perform CRRT.

## Discussion

While studies assessing general AKI clinical practices in China have been previously published, this survey is the first focused specifically on RRT practices. It was comprised equally of nephrologists and critical care physicians, the two sub-specialty groups almost exclusively responsible for managing acute RRT in China. The queried physicians, all of whom had some CRRT experience, represented a broad spectrum with regard to seniority, hospital type (Class II vs III), and practice location based on city size (Tier 1, 2, or 3). Finally, the survey data were obtained from 189 hospitals across 81 cities and 26 provinces in China. As such, we believe the survey is generally representative of RRT practices in China as a whole.

Reliable data estimating the incidence of RRT-requiring AKI in China do not currently exist. Our data indicate approximately eight AKI patients (mean value) developed an indication for RRT per hospital on a monthly basis and were most commonly treated in Class 3 hospitals, as evidenced by higher monthly RRT-requiring incidence rates there of approximately 10 and nine patients for nephrology and ICU patients, respectively. Of interest, CRRT machine availability on average was 65% greater in the nephrology department of this type of hospital versus the ICU (mean of 4.3 vs 2.6 machines, respectively). However, 90% of RRT-requiring patients treated in the ICUs of Class 3 hospitals initially received CRRT, approximately twice the percentage for such patients treated in a nephrology setting. Taken together, these data provide one line of evidence suggesting substantially higher CRRT utilization rates among critical care physicians than nephrologists.

The survey also demonstrated CRRT utilization is highest in Tier 2 cities, a somewhat surprising finding on first inspection. However, due to larger populations in Tier 1 cities, a relatively greater number of hospitals (both Class 2 and Class 3) is found in such areas versus Tier 2 cities. The relative scarcity of hospitals in Tier 2 cities, especially Class 3 hospitals having the highest CRRT utilization, results in a concentration of healthcare services (including CRRT) at Tier 2 hospitals. As such, CRRT capacity is actually higher at hospitals in Tier 2 cities.

In addition to the location of care and the prescribing physician, other clinical factors influenced RRT modality choice. Consistent with the KDIGO AKI Clinical Practice Guideline [**17**], the survey respondents designated hemodynamic status as a major consideration [**18**, **19**]. Likewise, fluid balance [**20**] was considered an important criterion, although Chinese physicians tolerated significant degrees of fluid overload before initiating RRT, irrespective of modality (see below).

Nevertheless, the survey data demonstrate the relationship between initial modality choice and hemodynamic status was unpredictable. The high percentage of hemodynamically *stable* patients initially treated with CRRT in the ICU setting suggests RRT allocation is dictated by local ICU policies at many Chinese hospitals. This approach is consistent with that used around the world by many ICUs in which critical care physicians manage RRT [**21, 22**]. On the other hand, a relatively large percentage of patients treated with conventional HD in the nephrology setting were hemodynamically *unstable*. This may be a cause for concern with the recognition that therapy is being delivered outside a critical care environment in this latter case.

It is important to note the questionnaire did not provide specific criteria for either AKI or hemodynamic instability and the survey participants were not required to provide such information in their responses. Furthermore, with respect to these two clinical characteristics, the possibility that different criteria may have been used by nephrologists and critical care physicians was not addressed by the survey.

The survey data related to RRT initiation [**23–25**] were also conflicting in several respects. Along with electrolyte/acid-base disturbances, multi-organ failure, and fluid overload, “acute kidney injury” was commonly reported as an important initiation criterion. Nevertheless, the majority of respondents did not believe azotemia is a critical factor. Second, while fluid management was considered in general to be an important criterion for RRT modality choice, fluid accumulation [**26**] did not appear to be a significant consideration when deciding upon the timing of RRT initiation. Nearly 95% of respondents either did not believe fluid overload (%FO) is a valid RRT initiation criterion or were willing to delay initiation of RRT until %FO exceeds 15%. (For the survey, the standard definition of %FO [**26**] was applied, with the assumption that the participants considered weight gain to be equivalent to fluid accumulation.) On the other hand, only 5% of respondents reported using a threshold %FO value of 10% as an initiation criterion. These results are surprising in light of the rapidly accumulating evidence base demonstrating an association between mortality and %FO values of greater than 10% at RRT initiation [**27–29**]. Finally, another obviously critical determinant of fluid balance in critically ill AKI patients, urine output [**30**], was prioritized by a substantial percentage of respondents, especially critical care physicians. Thus, the responses regarding RRT initiation criteria provide a mixed picture overall.

In some important respects, the survey responses indicate CRRT practices in China differ substantially from those in the developed world. Some of these differences are driven by the treatment location and prescribing physician. While the provision of CRRT in a nephrology department outside the ICU is very common in China, this practice is distinctly unusual worldwide–as far as we know, it does not occur systematically in any other country. The survey also revealed that another atypical CRRT practice in China is the prescription of less than 10 hours per day, especially in the nephrology-controlled environment. As discussed below, non-medical considerations may partly explain these differences.

On the other hand, the survey also indicates CRRT practices in China in many respects are quite similar to those in the developed world. Indeed, the survey responses reporting a wide variety of prescribed CRRT modalities [**31**], a prescribed hourly dose generally in the range of 20–30 mL/kg/hr [**17**], and an overall treatment duration generally in the 3 to 7-day range [**18, 21, 32–34**] are all consistent with worldwide CRRT practice. (However, effective dose delivered on a daily basis frequently falls below international standards due to relatively low treatment durations per day, as noted above.) Likewise, the prioritization of bleeding, filter clotting, and hypotension as complications is also consistent with prior reports on an international scale [**35**]. While the utilization of RCA in China lags somewhat behind the developed world [**36**], it is not evident whether this finding is due primarily to the lack of commercially available, citrate-containing replacement fluid and dialysate or physician preferences—the survey did not provide insight on this question.

Finally, it is important to put these results in the context of the current healthcare landscape in China. At present, hospital reimbursement and patient self-payment policies vary considerably across the country [**37**], resulting in the need for some patients and their families to make very difficult decisions about potentially life-saving medical technologies. The survey indicates 15% of Chinese AKI patients who are judged to be RRT candidates on clinical grounds ultimately do not receive dialysis. The decision to withhold treatment was largely attributed to the considerable out-of-pocket expenses that patients and their families considered untenable. Other factors considered important included patient frailty and advanced age, futility, and inadequate time or training for clinicians to perform RRT. However, it should be emphasized that these factors were not systematically assessed and were instead ascertained only from a small subset of survey participants. While the authors are not aware of published data regarding the estimated rate of withholding acute dialysis in the developed world, clinical experience suggests this is a rare occurrence.

Decisions to limit the daily duration of CRRT, especially in the nephrology setting, were likely influenced by considerations similar to those mentioned just above. Additional survey evidence that cost plays a significant role in modality selection is its designation as the foremost barrier to CRRT utilization. These cost-related factors also represent a divergence from the developed world, in which varying combinations of public and private health insurance coverage and generous reimbursement policies render these considerations relatively unimportant. While access to RRT is particularly limited in rural areas in China, it is improving in urban areas where incomes are higher and health insurance coverage is more extensive.

Although our study provides important insights about RRT practices for AKI in China, its limitations should be mentioned. The data generated from the survey were relatively qualitative in nature. Nevertheless, specific insights generated by our survey may lead to more quantitative studies in the future. Second, although there are clear differences in the manner in which Chinese AKI patients are treated in the ICU and nephrology settings, the latter is in general a more diverse clinical care environment. Specifically, patients ranging from those with AKI in the setting of multi-organ failure to others with much less complicated AKI (e.g., aminoglycoside toxicity) all may receive RRT in the nephrology setting. This heterogeneity may render problematic comparisons between ICU and nephrology patients and should be assessed in future studies. Finally, we are aware that considerable variation in RRT prescription and delivery still exists at the province, city, and hospital levels and acknowledge the survey data may not reflect Chinese clinical practice in all respects. Nevertheless, while worldwide RRT practices in AKI are certainly not entirely standardized yet [**38**], we believe the survey provides a reference point from which harmonization with worldwide RRT practices can continue [**39–41**].

## Conclusion

Our analysis demonstrates both similarities and differences between RRT practices for AKI in China and those in the developed world. Two differing aspects are particularly noteworthy. One is the relatively high percentage of RRT candidates who fail ultimately to receive renal support in China while the other is the unique dichotomy of care delivery based on prescribing physician and location of care. The latter difference is especially striking with regard to CRRT, as several aspects of nephrology-based management of this therapy in particular diverge from standard clinical practice. While some of these differences are driven by non-medical factors, future studies should explore these issues further to continue the process of harmonization of Chinese RRT practices with those in the rest of the world.

## Supporting information

S1 Table Questions used for survey of RRT practices in China. (DOCX)
