# Supplementary material for: Renal replacement therapy practices for patients with acute kidney injury in China
Source: PLoS One. 2017 Jul 10;12(7):e0178509. doi: 10.1371/journal.pone.0178509 (PMC5503167; doi:10.1371/journal.pone.0178509)
Supplement: S1 Table — (DOCX) [file pone.0178509.s001.docx]

**S1 Table: Questions used for survey of RRT practices in China**

1. Please choose the department in which you work：

- Nephrology
- ICU

1. Please choose your hospital class:

- Class 2
- Class 3

1. Please choose your duration of experience with prescribing CRRT

- < 1 year
- 1-3 years
- > 3 years

1. Please choose your professional title

- Resident
- Attending
- Associate chief physician
- Chief physician

1. Please provide your hospital name
2. Please provide the number of beds available for RRT in your department
3. Please provide the number of CRRT machines in your department
4. Please provide the monthly number of AKI patients who are considered RRT candidates in your department
5. Please provide the percentage of AKI patients who are RRT candidates but do not receive RRT in your department
6. Among patients who receive RRT in your department, please provide the percentage which is hemodynamically unstable for each modality:

Among patients treated with CRRT

Among patients treated with IHD

Among patients treated with SLED

Among patients treated with PD

1. Please choose the most common indication(s) for RRT in your department (choose 1-3 indications)：

- AKI
- Fluid overload
- Metabolic acidosis
- Electrolyte disturbance
- Sepsis
- MODS
- ARDS
- Crush syndrome
- Severe burn
- Severe pancreatitis
- Intoxication
- Others

1. What criteria are used to decide the timing of initiation of RRT in your department?

- Creatinine： □ >200umol/L，□ >300umol/L，□ >400umol/L, □Do not consider
- Urea： □ >10mmol/L，□ >24mmol/L，□ >41mmol/L, □Do not consider
- Potassium：□ >6.0 mmol/l，□ >6.5 mmol/l，□ >7.0 mmol/l, □Do not consider
- Urine output (<0.5ml/kg/h): □ > 6h，□ > 12h，□ > 24h, □Do not consider
- Fluid overload ：□ mild (FO>10%)，□moderate (FO >15%)，□ severe (FO>20%), □Do not consider
- Time from diagnosis to initiation of RRT：□<6h, □6-12h, □12-24h, □>24h, □Do not consider

1. What is the most commonly applied vascular access for RRT in your department?

- Jugular vein
- Femoral vein
- Subclavian vein
- Others

1. In your opinion, the main factors determining modality choice are：

Do not Possible Key consideration

- Hemodynamic status □ □ □
- Cytokine clearance □ □ □
- Fluid management □ □ □
- Preserving RRF □ □ □
- Clinical resource □ □ □
- Therapy cost □ □ □
- Clinical experience □ □ □
- Patient willingness □ □ □
- Others

1. What is the primary treatment mode of CRRT for patients in your department?

- CVVHD
- CVVH
- CVVHDF
- Others

1. What is the prescribed dose of CRRT in your department?

- < 25ml/kg/h
- 25–35 ml/kg/h
- >35 ml/kg/h
- Fixed dosage ml/h

1. The following types of anticoagulation are used in what percentage(s) of CRRT patients treated in your department?

- Heparin %
- Low molecular weight heparin %
- Regional citrate anticoagulation %
- Heparin-free %
- Others, %

1. What is the average total duration of CRRT in your department?

- < 3 days
- 3-7 days
- > 7 days

1. What is the average CRRT filter life in your department?

- < 12 hours
- 12–24 hours
- 24–36 hours
- > 36 hours

1. What is the actual daily duration of CRRT in your center on average?

- < 10 hours/day
- 10–20 hours/day
- 20–24 hours/day

1. Your degree of concern about the following CRRT complications is：

mild moderate strong

- Hypotension □ □ □
- Bleeding □ □ □
- Filter clotting □ □ □
- Catheter-related □ □ □
- Allergy to membrane □ □ □
- Drug clearance □ □ □

1. From your point of view, which of the following is (are) the most important CRRT-related factor(s) influencing survival? (Please choose 1-3 options)

- Timing of CRRT initiation
- CRRT dosage
- CRRT modalities
- Anticoagulation type
- Membrane material and filter efficacy
- Fluid management
- Drug dosage modification and nutritional support
- Others,

1. From your point of view, which of the following is (are) the most important CRRT-related factor(s) influencing renal recovery? (Please choose 1-3 options)

- Timing of CRRT initiation
- CRRT dosage
- CRRT modalities
- Anticoagulation type
- Membrane material and filter efficacy
- Fluid management
- Drug dosage modification and nutritional support
- Others,

1. In your department, follow-up of AKI survivors is best described as：

- Regular follow-up of primary diseases
- Follow-up by nephrologists
- No specific follow-up

1. From your point of view, what are the major challenge(s) that CRRT presents? (Please choose 1-3 options)

- Lack of clinical effectiveness index
- Lack of defined criteria for timing of CRRT initiation
- Lack of therapy standardization
- Lack of specific training requirements for CRRT qualification
- Lack of doctors and nurses with competence to perform CRRT
- Affordability for patients
- Others,
